# Professional Secrets

**Published:** 1883-09-20

**Authors:** 


### *PROFESSIONAL SECRETS.*

The true and honorable physician will not, under any circumstances, disclose the secrets of his patient. The public and the courts approve and recognize this duty of the practitioner. In a recent decision of the Supreme Court of Missouri, it was held that a physician could not be compelled, on the witness' stand, to disclose a fact obtained by diagnosis of the case, even where the patient had not specially enjoined confidence, it being now well understood and confirmed by common consent and usage, that the physician will not disclose the secrets of his patients,

